# Enrichment and characterization of cancer stem-like cells from a cervical cancer cell line

**DOI:** 10.3892/mmr.2014.2063

**Published:** 2014-03-20

**Authors:** LI WANG, HUIJIE GUO, CAIYU LIN, LIUQI YANG, XIUJIE WANG

**Affiliations:** Laboratory of Experimental Oncology, State Key Laboratory of Biotherapy, West China Hospital, West China Clinical Medical School, Sichuan University, Chengdu, Sichuan 610041, P.R. China

**Keywords:** cervical cancer, cancer stem cells, nonadhesive culture, cancer stem cell enrichment, chemoresistance, differentiation

## Abstract

Cancer stem cells (CSCs) are proposed to be responsible for tumor recurrence, metastasis and the high mortality rate of cancer patients. Isolation and identification of CSCs is crucial for basic and preclinical studies. However, as there are currently no universal markers for the isolation and identification of CSCs in any type of cancer, the method for isolating CSCs from primary cancer tissues or cell lines is costly and ineffective. In order to establish a reliable model of cervical cancer stem cells for basic and preclinical studies, the present study was designed to enrich cervical cancer CSCs using a nonadhesive culture system and to characterize their partial stemness phenotypes. Human cervical cancer cells (HeLa) were cultured using a nonadhesive culture system to generate tumor spheres. Their stemness characteristics were investigated through colony formation, tumor sphere formation, self-renewal, toluidine blue staining, chemoresistance, invasion assays, reverse transcription-polymerase chain reaction, immunofluorescence staining of putative stem cell markers, including octamer-binding transcription factor 4, SRY-box 2 and aldehyde dehydrogenase 1 family, member A1, and adipogenic differentiation induction. Typical tumor spheres were formed within 5–7 days under this nonadhesive culture system. Compared with the adherent parental HeLa cells, the colony formation capacity, self-renewal potential, light cell population, cell invasion, chemoresistance and expression of putative stem cell markers of the tumor sphere cells increased significantly, and a subpopulation of tumor sphere cells were induced into adipogenic differentiation. Using the nonadhesive culture system, a reliable model of cervical cancer stem cells was established, which is inexpensive, effective and simple compared with the ultra-low attachment serum free culture method. The stemness characteristics of the tumor sphere HeLa cells mirrored the CSC phenotypes. This CSC model may be useful for basic and preclinical studies of cervical cancer and other types of cancer.

## Introduction

Cervical carcinoma is the second most common type of malignant cancer and the fourth leading cause of cancer-associated mortality in females worldwide (1). Currently, surgery, chemoradiotherapy, HPV vaccines and associated biological therapy are the main modalities for the treatment of cervical cancer; however, they all have limitations. The HPV vaccines are only effective for HPV types 16 and 18, however, there are other high-risk subtypes that are also able to cause cervical cancer (2). Patients with cervical carcinoma undergoing surgery and chemoradiotherapy have a survival advantage (3), however, data suggested that the recurrence incidence of cervical cancer diagnosed in females accounts for ~35%, and 90% were found within three years after the initial management (4). It was demonstrated that certain cervical cancer cells were not eradicated by current therapeutics.

The cancer stem cell (CSC) theory provided novel insights into the recurrent formation of tumors following surgery or chemoradiotherapy in cancer patients. CSCs possess certain properties, including a capacity for self-renewal, chemoresistance, the ability to differentiate into mature, specialized cancer cell types as well as a high tumorigenic potential that may correlate with the initiation, progression and recurrence of cancer (5). CSCs have been reported in multiple types of solid tumor and in cultured cancer cell lines, including brain (6), breast (7), colon (8) and prostate (9), as well as cervical cancer cell lines (10). Almost all the cancer stem-like cells have been isolated and cultured in serum-free medium supplemented with adequate mitogens, including basic fibroblast growth factor and epidermal growth factor, and incubated for 2–6 weeks, which is costly, time-consuming and ineffective (11–13). Recently, a novel method for overcoming these drawbacks and limitations, termed the nonadhesive culture system, was used to successfully isolate and enrich CSCs from established human oral squamous cell carcinoma (OSCC) cell lines (14). To establish a reliable *in vitro* model of CSCs of cervical cancer for basic and preclinical studies, the present study was designed to enrich and identify stem-like cells from human cervical cancer cells (HeLa), and to further characterize their CSC properties.

## Materials and methods

### Cell line and culture

The human cervical cancer cell line, HeLa was obtained from the Shanghai Cell Biology Institute of the Chinese Academy of Sciences (Shanghai, China). The parental adherent monolayer HeLa cells were maintained in Dulbecco’s modified Eagle’s medium (DMEM) supplemented with 10% fetal bovine serum (FBS), penicillin (100 U/ml) and streptomycin (100 μg/ml) in a humidified atmosphere of 50 μg/ml CO_2_ at 37°C.

### Tumor sphere culture

The tumor spheres of HeLa cells were cultured using the nonadhesive culture system described by Chen *et al* (14) with minor modifications. Briefly, the parental adherent monolayer HeLa cells were collected and plated in 100-mm dishes coated with agarose at a density of 5×10^4^ cells, and the culture medium was altered every other day until tumor spheres were formed.

### Colony formation assay

The colony forming ability of the parental adherent monolayer and tumor sphere HeLa cells were assayed by replating them in 6-well plates (200 cells/well). Following 12 days of incubation, the cells were stained with 0.5% crystal violet in absolute ethanol, and colonies with >50 cells were counted under a dissection microscope [Olympus (China) Co., Ltd., Beijing, China]. Three independent experiments were performed.

### Tumor sphere formation and self-renewal assay

The tumor spheres were collected by gentle centrifugation, disaggregated with Accutase (Sigma-Aldrich, St. Louis, MO, USA) to generate single cells and passaged every 5–7 days when the spheres reached a diameter of 100 μm. To evaluate tumor sphere forming efficiency, single tumor sphere cells derived from the parental or tumor spheres were plated into 96 wells at varying densities; the lowest density was one cell per well. Following 12 days of culture, the sphere number of each well was counted. Sphere forming efficiency was calculated as the sphere number divided by the initial single cell number plated and expressed as a percentage (15). In addition, the wells with only one cell were monitored. The spheres derived from single cells were marked and images of the spheres were captured every day.

### Toluidine blue staining

To evaluate the light cell (LC) and dark cell (DC) populations in the parental adherent monolayer and tumor sphere HeLa cells, the two cell suspensions were stained with toluidine blue staining buffer containing 10 mM HEPES buffer (pH 7.4), 2 mM EDTA, 0.5% bovine serum albumin (BSA) and 0.4% toluidine blue (Sigma-Aldrich) for 5 min at room temperature (RT) (7). Images of the cells were captured with a photocamera-equipped light microscope [Olympus (China) Co., Ltd.]. An average of six fields/sample was analyzed and three independent experiments were performed.

### Chemotherapy sensitivity and resistance assays

The chemoresistance of the parental adherent monolayer and tumor sphere HeLa cells was assessed using a modified MTT assay (16). Briefly, 2×10^3^ cells per well were seeded in 96-well plates in 100 μl culture medium (three wells per group). Following 24 h, the cells were treated with various concentrations of cisplatin and epirubicin, respectively, for 72 h. Subsequently, 10 μl MTT solution was added to each well and the plate was incubated in the dark for an additional 4 h at 37°C. The cells were then lysed in a buffer containing 10% sodium dodecyl sulfate in 0.01 M HCl. The absorbance at 570 nm was measured using a microplate reader (Bio-Rad, Richmond, CA, USA), using wells without cells as blanks. The effects of cisplatin and epirubicin on the viabilities of adherent monolayer and tumor sphere HeLa cells were expressed as the %cytoviability using the following formula: %cytoviability = A_570_ of treated cells/A_570_ of control cells × 100% (17). Three independent experiments were performed.

### Invasion assay

The invasion assay was performed using 24-transwell chambers (Costar, Bodenheim, Germany). Briefly, the parental adherent monolayer and tumor sphere HeLa cells were resuspended in serum-free DMEM at a concentration of 4×10^5^ cells/ml. The upper chamber was loaded with 100 μl cell suspension and the lower chamber was loaded with 500 μl DMEM with 15% FBS. Following culture for 48 h, the cells in the upper chamber were removed using a cotton swab and the lower chamber filter was fixed with 4% paraformaldehyde and stained with crystal violet. The number of cells that migrated to the undersurface of the membrane was counted and six randomly selected fields were analyzed. Three independent experiments were performed.

### Western blotting analysis of Oct4 and Sox2 protein expression

The proteins of the parental adherent and tumor sphere cells were prepared with RIPA lysis buffer (Beyotime Institute of Biotechnology, Shanghai, China), separated by 12% SDS-PAGE, transferred onto PVDF membranes using a semi-dry blotting apparatus (Bio-Rad, Hercules, CA, USA), and blocked in 5% non-fat milk. The membranes were subsequently incubated with the corresponding primary antibodies, as indicated: a rabbit anti-β-actin (Beijing Biosynthesis Biotechnology Co., Ltd., Beijing, China) diluted 1:300, rabbit anti-Oct4 and rabbit anti-Sox2 (BioLegend, San Diego, CA) diluted 1:1,000. Antibody recognition was detected with peroxidase-conjugated goat anti-rabbit IgG (H+L) secondary antibody (Zhongshan Goldenbridge Biotechnology Co., Ltd, Beijing, China) used at 1:3,000 dilutions. Antibody-bound proteins were detected with a BeyoECL Plus kit (Beyotime Institute of Biotechnology) and western blotting analysis system (Universal Hood II, Bio-Rad, USA ).

### Detection of aldehyde dehydrogenase 1 (ALDH1) and SOX2 by immunofluorescence staining

The parental adherent monolayer and tumor sphere HeLa cells were fixed with 4% paraformaldehyde for 10 min, permeabilized with 0.01% Triton X-100 and inhibited with 5% BSA in phosphate-buffered saline (PBS). The cells were then incubated with rabbit anti-ALDHA1-fluorescein isothiocyanate (FITC) polyclonal antibody (Beijing Biosynthesis Biotechnology Co., Ltd., Beijing, China) and rabbit anti-SOX2-FITC (Epitomics, Inc., Burlingame, CA, USA) in 1% BSA and PBS with Tween-20 in a humidified chamber for 1 h at RT, respectively. DAPI (Sigma-Aldrich) was used for nuclear counterstaining. Images were captured using a Leica DMI400B inverted fluorescence microscope linked to a DFC340FX camera (Leica Microsystems GmbH, Wetzlar, Germany). Three independent experiments were performed.

### Adipogenic differentiation assay

For the adipogenic differentiation assay, tumor sphere HeLa cells were seeded in 6-well plates, cultured with DMEM containing 5% FBS, supplemented with 10 μM insulin, 1 μM dexamethasone, 200 μM indomethacin and 3-isobutyl-1-methylxanthine (Sigma-Aldrich). The culture medium was altered twice a week and the appearance of lipid droplets was monitored every day. Following incubation for 15 days, the medium was aspirated and the cells were washed with PBS and fixed with 4% paraformaldehyde in PBS for 30 min. Then, the cells were incubated with Oil Red O dye (Sigma-Aldrich) at RT for 30 min. The dye was carefully removed, washed with PBS and counterstained with hematoxylin. Images were captured using a Leica DMI400B inverted fluorescence microscope linked to a DFC340FX camera (Leica Microsystems GmbH).

### Statistical analysis

The data are expressed as the mean ± standard deviation. All data were analyzed using the software SAS V9.1 (SAS Institute Inc., Cary, NC, USA). Student’s t-test was used to analyze the statistical difference. P<0.05 was considered to indicate a statistically significant difference.

## Results

### Morphological characteristics

**Figure 1:**
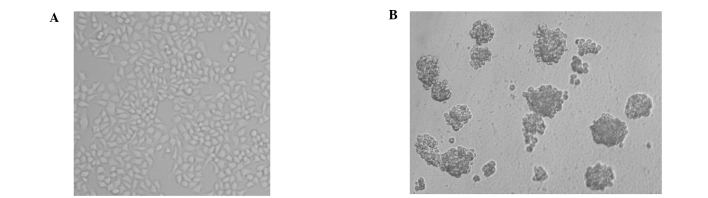
Morphology of the parental adherent monolayer and tumor sphere HeLa cells. (A) Parental HeLa cells cultured in Dulbecco’s modified Eagle’s medium + 10% fetal bovine serum grew as an adherent monolayer. (B) Tumor sphere HeLa cells derived from the parental HeLa cells cultured under a nonadhesive culture system formed typical tumor spheres.

The parental HeLa cells cultured with DMEM supplemented with 10% FBS grew as an adherent monolayer (Fig. 1A), while HeLa cells cultured under the nonadhesive culture system formed typical tumor spheres (Fig. 1B).

### Tumor sphere cells exhibit a higher colony forming efficiency compared with parental adherent monolayer cells

**Figure 2:**
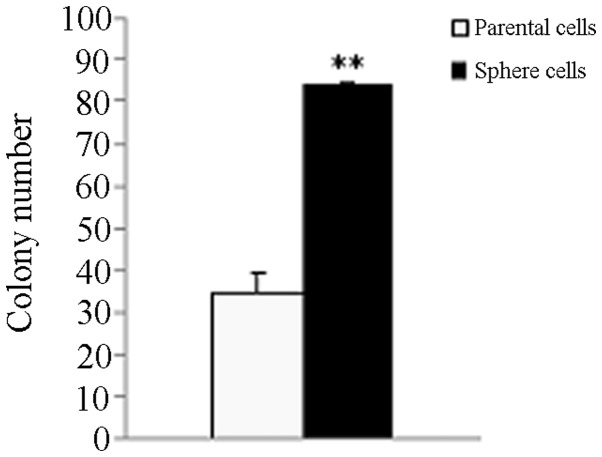
Colony formation of parental and tumor sphere HeLa cells. Parental and tumor sphere HeLa cells were seeded onto 6-well plates at 300/well. The colony number was counted under a dissection microscope. ^**^P<0.01, vs. control. The results are expressed as the mean ± standard deviation of three independent experiments.

The parental HeLa cells generated 34.67±4.51 colonies and the tumor sphere cells generated 83.67±8.50. The tumor sphere cells exhibited a higher colony forming efficiency compared with the parent adherent monolayer cells (P<0.01; Fig. 2).

### Tumor sphere cells exhibit a high self-renewal potential

**Figure 3:**
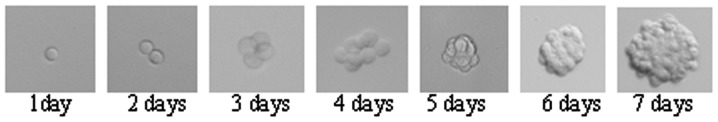
Tumor sphere formation process from a single HeLa cell tumor sphere.

The sphere formation assay has been universally used to evaluate the property of progenitors or stem cells. The parental HeLa cells grew as an adherent monolayer in DMEM containing 10% FBS (Fig. 1A). When plated in an agarose coated nonadhesive culture system, they grew as floating, three-dimensional tumor spheres and reached 100 μm in diameter following 7 days (Fig. 1B). Tumor spheres were passaged and plated into 96 wells at varying densities; the lowest density was one cell per well. Following 12 days of culture, the sphere formation efficiency of tumor sphere HeLa cells was 40.79±1.8% (data not shown). Tumor sphere formation from single cells was observed (Fig. 3).

### Toluidine blue pale LC and DC populations in the parental adherent monolayer and tumor sphere HeLa cells

**Figure 4:**
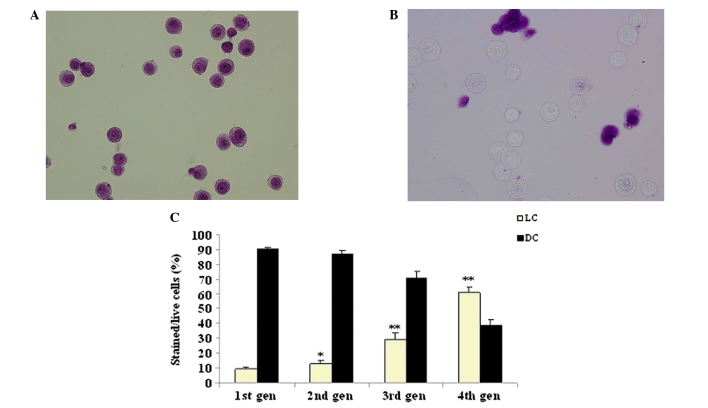
Toluidine blue LC and DC cell subpopulations in the parental adherent monolayer and tumor sphere HeLa cells. (A) Toluidine blue pale (LC) and dark (DC) cell subpopulations in the parental adherent monolayer HeLa cells. (B) Toluidine blue LC and DC cell subpopulations in the tumor sphere HeLa cells. Magnification, ×200 (C) Histogram indicating that there was a significant increase in the toluidine blue LC cell subpopulation and a decrease in the DC cell subpopulation in tumor sphere HeLa cells between passage one and passage four. ^*^P<0.05 and ^**^P<0.01, vs. control. The results are presented as the mean ± standard deviation of three independent experiments. LC, light cell; DC, dark cell.

The parental adherent monolayer and tumor sphere HeLa cells contained distinct LC and DC populations (Fig. 4A and B). The number of LCs in tumor sphere HeLa cells (60.94%) was higher than those in the parental adherent monolayer HeLa cells (2.2%; Fig. 4B; P<0.01). LC populations in the tumor sphere increased gradually between 9.48+0.9 and 60.94+3.2% (Fig. 4C) following four passages.

### Tumor sphere HeLa cells are resistant to chemotherapy compared with parent adherent monolayer HeLa cells

**Figure 5:**
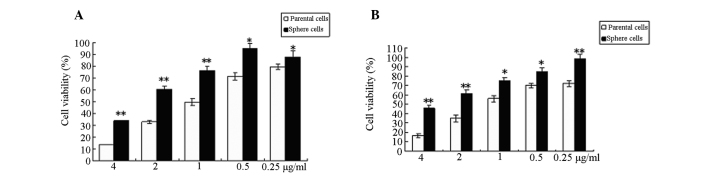
Cell viability assays of the parental adherent monolayer and tumor sphere HeLa cell response to cisplatin and epirubicin. (A) Cell viability assays of the parental adherent monolayer and tumor sphere HeLa cell response to cisplatin. (B) Cell viability assays of the parental adherent monolayer and tumor sphere HeLa cell response to epirubicin. ^*^P<0.05 and ^**^P<0.01, vs. control. The results are expressed as the mean ± standard deviation of three independent experiments.

To assess the chemoresistance of the parental adherent monolayer and tumor sphere HeLa cells, the two cell populations were treated with cisplatin and epirubicin, respectively for 48 h. The viability of tumor sphere HeLa cells was higher than adherent monolayer HeLa cells at the same concentration of the drug (P<0.05 or P<0.01; Fig. 5A and B).

### Tumor sphere cells exhibit a high invasive capacity

**Figure 6:**
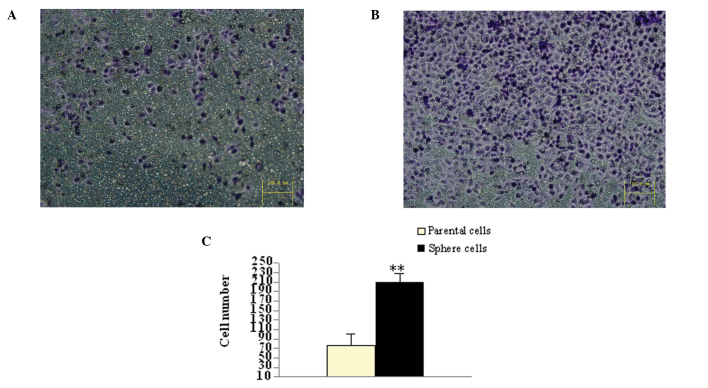
Invasion assay of the parental adherent monolayer and tumor sphere HeLa cells. (A) Invasive cells of the parental adherent monolayer HeLa cells. (B) Invasive cells of tumor sphere HeLa cells. (C) Histogram indicating that there was a significant increase in invasive cells of the tumor sphere HeLa cells. ^**^P<0.01, vs. control. The results are expressed as the mean ± standard deviation of three independent experiments.

The invasive capacity of the parental adherent monolayer and tumor sphere HeLa cells were determined using a transwell invasion assay. Following incubation for 48 h, the number of cells that penetrated the transwell membrane (208±18/field; magnification, ×200) of tumor spheres was higher than the parental adherent monolayer HeLa cells (75±24/field; magnification, ×200; P<0.01; Fig. 6).

### Protein expression of Oct4 and SOX2 increases in tumor sphere HeLa cells compared with the parental adherent monolayer

**Figure 7:**
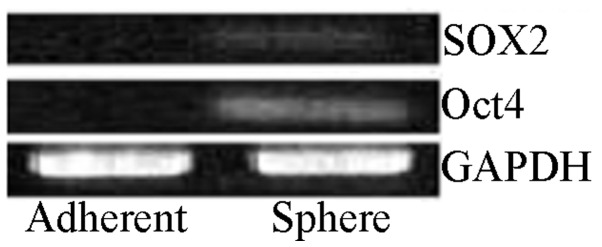
Protein expression of Oct4 and SOX2 in tumor sphere HeLa cells. OCT4, octamer-binding transcription factor 4; SOX2, SRY-box 2.

Western blotting demonstrated that there was little expression of Oct4 and SOX2 in the parental adherent monolayer HeLa cells, while their expression was evident in tumor sphere HeLa cells cultured under a nonadhesive culture system (Fig. 7).

### Tumor sphere HeLa cells express high levels of the putative stem cell markers, ALDH1 and SOX2

**Figure 8:**
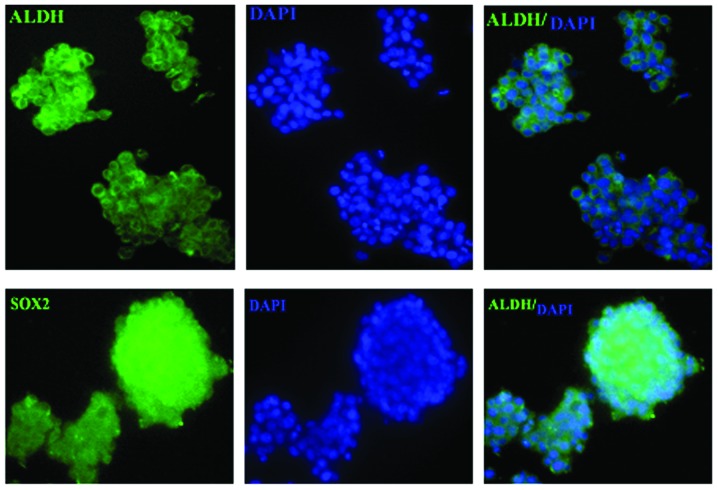
Tumor sphere HeLa cells expressed high levels of the putative stem cell markers, ALDH and SOX2, which were detected with fluorescein isothiocyanate-labeled ALDH and SOX2 polyclonal antibodies. Green, expression levels of ALDH and SOX2; blue, DAPI-stained cell nuclei. SOX2, SRY-box 2; ALDH, aldehyde dehydrogenase.

To detect the expression of putative CSC markers in tumor sphere HeLa cells, the parental adherent monolayer and tumor sphere HeLa cells were examined for ALDH1 and SOX2 protein expression. The parental adherent monolayer HeLa cells scarcely expressed ALDH1 and SOX2, while stable ALDH1 and SOX2 expression was detected in tumor sphere HeLa cells (Fig. 8).

### Tumor sphere HeLa cells are induced to differentiate into adipocytes

**Figure 9:**
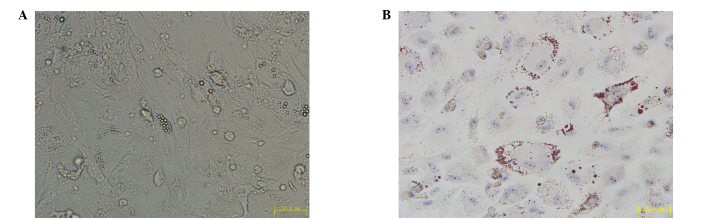
Adipogenic differentiation of tumor sphere HeLa cells. (A) Lipid droplets present in the cytoplasm. (B) Lipid droplets were confirmed with Oil Red O staining (magnification, ×400; scale bar, 50 μm).

To determine the multipotent differentiation potential of the tumor sphere cells, the tumor sphere HeLa cells were cultured in adipogenic differentiation media for 15 days and small lipid droplets were observed in the cytoplasm of cancer cells. The lipid droplets increased in size with time (Fig. 9A) and were confirmed by Oil Red O staining (Fig. 9B).

## Discussion

Cervical carcinoma, a prevalent disease, is considered to be the second most common type of malignant cancer and the fourth leading cause of cancer-associated mortality in females worldwide (1). Cervical cancer has a high recurrence rate and high risk of metastasis following conventional therapy, leading to a high mortality rate (2–4). Previous studies indicated that a small population of CSCs appear to be responsible for tumor initiation and progression and also for the resistance to conventional treatment (18,19). Recently, isolation, identification and selective eradication of CSCs using targeted drugs have become a major focus in basic and clinical cancer studies (20,21). Therefore, for investigating and developing agents targeting CSCs for cancer therapeutics, a reliable model of CSCs is crucial for basic and preclinical studies. However, there are currently no universal markers for the isolation and identification of CSCs in any type of cancer (14). Cell lines cultured with defined serum-free culture conditions are a commonly used method for enriching CSCs from mixed populations and has been particularly important in establishing *in vitro* models for CSC expansion (22). However, it is costly, time-consuming and ineffective (11–13).

Using the nonadhesive culture method to enrich CSCs from the human OSCC cell line was cost-effective and simple (14).

In the present study, human cervical cancer stem-like cells were enriched and expanded using a nonadhesive culture system and the majority of cells formed typical tumor spheres (Fig. 1B). The colony and tumor sphere formation efficiency of cancer stem-like cells from tumor spheres was higher than the parental adherent monolayer HeLa cells (Fig. 2; P<0.01). Single cells derived from tumor spheres were able to generate the second tumor spheres (Fig. 3), which reflects the self-renewal potential of cancer stem-like cells. The cancer stem-like cells from tumor spheres stained pale with toluidine blue (LCs; Fig. 4) and were endowed with features of CSCs (7). Stemness-associated genes Oct4 and SOX2 and putative stem cell markers, ALDH and Oct4 were expressed in the tumor sphere cells but not in the parental adherent monolayer HeLa cells.

Positive stemness markers and the ability to form spheres are considered to be hallmarks of CSCs (7,14,22,23), which are endowed with chemoresistance (18,19) and a high invasive capacity (24). In the present study, the cancer stem-like cells from tumor spheres were more resistant to cisplatin and epirubicin (Fig. 5) and exhibited a higher invasive potential (Fig. 6) than the Bcrp1-positive cervical CSCs (25). These findings demonstrated that the tumor sphere cells cultured in this nonadhesive culture system exhibited stemness (18,19,24,25). The findings in the present study suggested that the cancer stem-like cells that were enriched and expanded under this experimental condition may be useful for basic and preclinical studies of cervical CSCs and or other solid CSCs.

Inducing the differentiation of CSCs, aimed at attacking the stemness of CSCs and reducing their chemo and radioresistance, represents a novel modality for cancer stem-cell-targeting therapy (26). Adipogenic differentiation induction of cancer cells was previously reported in breast cancer cells (27–29) and prostate cancer cells (30); however, to the best of our knowledge, no studies have investigated the adipogenic differentiation induction of cervical CSCs. In the present study, adipogenic differentiation was induced in tumor sphere HeLa cells. This suggested that the stemness phenotype of cervical CSCs was able to be reversed and highlights a promising avenue for the therapeutics of cervical cancer through differentiation induction of CSCs.

In conclusion, the cervical cancer stem-like cells were enriched and expanded using a nonadhesive culture system. The enriched cancer stem-like cells exhibited the CSC phenotype and may be a useful model for investigating and developing substances targeting CSCs for the basic and preclinical investigations of therapeutics of cervical cancer and/or other types of solid cancer. The stemness phenotype of cervical CSCs was able to be reversed and the differentiation induction of cervical CSCs may be a novel modality in the treatment and/or prevention of human cervical cancer, and thus requires further investigation.
